# Photovoltaic Power Generation Forecasting Using a Novel Hybrid Intelligent Model in Smart Grid

**DOI:** 10.1155/2022/7495548

**Published:** 2022-10-06

**Authors:** Alexandre Teplaira Boum, Vinny Junior Foba Kakeu, Camille Franklin Mbey, Felix Ghislain Yem Souhe

**Affiliations:** Department of Electrical Engineering, University of Douala ENSET, Douala, Cameroon

## Abstract

The exponential growth of electrical demand and the integration of renewable energy sources (RES) brought new challenges in the traditional grid about energy quality. The transition from traditional grid to smart grid is the best solution which provides necessary tools and information and communication technologies (ICT) for service enhancement. In this study, variation of energy demand and some factors of atmospheric change are considered to forecast production of photovoltaic energy that can be adapted for evolution of consumption in smart grid. The contribution of this study concerns a novel optimized hybrid intelligent model made of the artificial neural network (ANN), support vector machine (SVM), and particle swarm optimization (PSO) implemented for long term photovoltaic (PV) power generation forecasting based on real data of consumption and climate factors of the city of Douala in Cameroon. The accuracy of this model is evaluated using the coefficients such as the mean square error (MSE), root mean square error (RMSE), mean absolute percentage error (MAPE), mean absolute error (MAE), and regression coefficient (R). Using this novel hybrid technique, the MSE, RMSE, MAPE, MAE, and *R* are 14.9721, 3.8693, 3.32%, 0.867, and 0.9984, respectively. These obtained results show that the novel hybrid model outperforms other models in the literature and can be helpful for future renewable energy requirements. However, the convergence speed of the proposed approach can be affected due to the random variability of available data.

## 1. Introduction

Nowadays, much of the world's electricity production depends on fossil fuel sources such as coal, gas, oil, and fuel [1]. The combustion of these materials causes environmental damage with the emission of carbon dioxide into the atmosphere [2]. The interest is therefore to have a reliable, intelligent, sufficient, robust, safe, efficient, and climate-clean electricity grid [3]. To this end, special and important attention has been paid to renewable energies which provide green electrical energy without emission of toxic gas to the atmosphere [4]. Distributed renewable energy generations have become emerging technologies with rising of renewable energies and the support provided by governments [5]. However, renewable energies such as solar energy and wind power show significant limits because of the perpetual variability of climate. In addition, the distribution of this energy is inadequate with the current network, these renewable energies produce variations and fluctuations which lead to instability of the network, and this is the main challenge when integrating renewable energy resources [6]. The technology of the current infrastructures existing in the network does not allow a correct integration of these renewable energies because of the requirements which they imply. It is therefore necessary to carry out a substantial planning with methods of investigation in the field to have the parameters of availability and compatibility of these renewable energies on the distribution network [7].

With the increasing penetration of renewable energies, major improvements and modifications must be made to the existing power electrical grid to ensure good accommodation with these intermittent resources [8]. Considering these requirements, it is vital to migrate to a smart grid using communication systems, a large network of sensors, and also an intelligent management system [9, 10]. So, with these assets, the electricity network will obtain a self-healing capacity, a correct integration of renewable energies, and a consequent efficiency [11, 12]. To face these new requirements in the grid, some countries have embarked on the transition to the smart grid technologies [13, 14]. The main objective of a smart grid is to increase energy efficiency, minimize energy demand, and reduce energy production costs [15]. It also ensures a stable system that cannot be affected by network failures [16]. The renewable energy sources that can be used are solar, hydraulic, geothermal, or biomass energies [17]. The smart grid can improve and facilitate operations on the network, allowing management of consumption data using smart meters [18]. In addition, network security is perfectly ensured with the smart grid privacy system. The integration of renewable energies is also ensured by the smart grid for a clean grid and green energy [19].

It is known that renewable energy sources bring enormous benefits to the electrical grid. However, they lead to major challenges in the structure of the smart electrical grid. Considering that these renewable sources depend on the climate, it is therefore necessary to forecast the power energy generation in order to maintain the stability of the network and ensure efficient management of production and the power produced by other energy sources [20].

The real world faces different levels of variability in renewable energy sources due to intermittent weather conditions. These uncertain energy sources can affect the forecasting of energy generation. In the literature, authors worked on methods which take into account these source intermittences and provide a better time series forecast of the power generation in the network [21, 22]. Author [23] reviewed the techniques of machine learning (ML) for the integration and forecasting of renewable energy sources. The main problem is to properly integrate renewable energy sources into the smart grid; it is therefore necessary to predict the amount of power that will be generated in the future. Knowing that the energy storage of solar panels is expensive, efficient management of this energy is necessary. This work offered different strategies for this power generation forecasting. Author [24] also reviewed deep learning (DL) for renewable energy forecasting. In addition, author [25] proposed a model of the uncertainties of renewable energy sources for the study of smart grid vulnerability. This model was applied to identify the dynamic uncertainties of renewable energy sources in order to reveal their characteristics in the frequency domain. This approach has been validated on an IEEE 300 bus network to assess the impacts of these uncertainties on power flow.

Some methods of probabilistic nature using historical data to define the density probability function to characterize the uncertainties of renewable energy sources have been proposed in [26]. Authors [27] studied the problem of energy management with various renewable energy sources. The resolution of this problem is done through the algorithm which simultaneously optimizes the load and the energy discharge in the storage units. Authors [28] explored forecasting of short-term generation of photovoltaic plants in a smart grid. In this article, some climatic factors are proposed to forecast the photovoltaic power generation using real-time data from solar plants located in Konya, Turkey. The authors used artificial neural networks (ANNs) implemented on MATLAB to forecast this photovoltaic generation. The artificial neural network algorithms such as Levenberg–Marquardt and Bayesian regularization are used to compare the forecasting results. In order to increase accuracy, authors [29] combined neural networks with a wavelet method. To perfectly ameliorate the forecasting accuracy, authors [30] also combined the machine learning technique with the neural networks.

Another technique of combining the neural network with an analogous ensemble method has been proposed in [31]. Authors [32] proposed a site-specific prediction model for photovoltaic power generation using climate parameters. In this study, authors used machine learning techniques as multiple regression techniques and least-square support vector machine (LSSVM). In the same time, authors [33] explored an intelligent predictor for 6 hours ahead solar power prediction. A combination method has been proposed in [34]. In this study, the authors proposed a combination of spatial modelling with neural networks. These combined methods unfortunately do not show great accuracy in forecasting results, and this can turn out to be a major drawback in renewable energy forecasting.

Authors [35] adopted a forecasting of photovoltaic solar energy for energy management in a smart grid. Since forecasting accuracy is vital for the safety and operation of the smart grid, this article reviewed theoretical forecasting methodologies for solar photovoltaic resources. In addition, applications of photovoltaic power generation forecasting are also detailed in this study. Hybrid artificial intelligence methods using neural networks are also presented in [36]. In fact, some approaches depend on forecasting levels to satisfy forecasting requirements [37]. Similarly, optimization algorithms and artificial intelligence techniques are used for forecasting photovoltaic generation in the smart grid [38]. However, optimizing these parameters does not ensure a significant improvement in accuracy as it negatively affects key assumptions of the neural algorithm.

Others works have also been proposed for the management, simulation, control, and modelling of renewable energy sources based on artificial intelligence and time series forecasting [39–41]. In the same way, authors [42] applied the Markov model using the hidden optimized algorithm for short-term prediction of photovoltaic power. In another work, authors [43] implemented the multidirectional search optimization algorithm for the tracking of photovoltaic energy systems. Obviously, these methods are very limited when the database is large and complex. Authors [44] developed a multihead convolutional neural network (CNN) in efficient renewable power sources for energy forecasting with the energy storage system. In addition, authors [45] proposed the adaptive neuro fuzzy inference system (ANFIS) based model to forecast solar energy under different weather conditions such as clear sky, hazy sky, and cloudy sky. This work is also proposed for smart grid applications in short-term forecasting of photovoltaic power.

In [46], a robust artificial intelligence (AI) algorithm is applied for power density prediction with wind power plant installation. In this study, the effects of the wind speed, the wind direction, the temperature, the damp, and the pressure on power density could be modelled. The obtained results show that the applied method is superior to traditional AI-based techniques. Authors [47] proposed a day ahead and 1 h ahead mean photovoltaic (PV) output power forecasting model based on the extreme learning machine (ELM) approach. Therefore, the developed model is trained and tested using power output of the photovoltaic system and other meteorological parameters recorded in three grid-connected PV system installed on a roof-top of PEARL Laboratory in University of Malaya, Malaysia. The performance of the proposed model is proven using statistical error indicators such as the root mean square error (RMSE), mean absolute percentage error (MAPE), mean absolute bias error (MABE), and coefficient of determination (*R*^2^). The comparison of results showed that the ELM model gives higher accuracy and less computational time in daily and hourly PV output power forecasting.

Moreover, authors [48] investigated the prediction of monthly and weekly wind power density of four different locations in Malaysia using the ANFIS model, the ANFIS-PSO model, the ANFIS-GA model, and the ANFIS-DE model. Author used statistical parameters such as the mean absolute bias error (MABE), mean absolute percentage error (MAPE), root mean square error (RMSE), and coefficient of determination (*R*^2^) to evaluate the performance of the proposed hybrid ANFIS models. Authors [49] developed statistical and neural network-based approaches to predict hourly wind speed data of the subsequent year. For this purpose, authors used a set of recent wind speed measurement samples from two meteorological stations in Malaysia. The obtained results by the proposed approach are rather promising in view of the very small mean absolute error (MAE). In addition, authors [50] used a new hybrid swarm technique to forecast the energy output of a real wind farm located in Binaloud, Iran. This hybrid method is made of ant colony optimization (ACO) and particle swarm optimization (PSO) which are two swarm intelligence meta-heuristic techniques. The obtained results show the effectiveness of the hybrid forecasting model with a faster convergence profile and a MAPE of 3.513%.

In this study, the authors proposed a novel hybrid intelligent model using the artificial neural network (ANN) and the support vector machine (SVM) in order to predict the energy production of solar panels considering climatic factors and the regular consumption of customers in order to adapt this power generation to consumption with the aim of ensuring a permanent supply of energy to consumers. Moreover, particle swarm optimization (PSO) was used in order to select suitable and optimal parameters for the SVM and ANN models. To our knowledge, this method has never been done in the past because its effectiveness is proven and applicable to any type of data, even climatic, economic, social, or environmental.

The structure of this study is presented as follows: Section 2 gives the materials and the methods used in this study including the ANN model, SVM model, and PSO algorithm for the forecasting. In Section 3, an implementation of this model is proposed to forecast photovoltaic power generation and obtain the accuracy results; moreover, the discussion of these results helps to show the effectiveness of new model.  Section 4 concludes this study and presents directions for the work in the future.

## 2. Materials and Methods

### 2.1. Materials

#### 2.1.1. Data Collected

First, to build the neural network approach, it is necessary to collect the data that will allow the model to be trained and give it enough information to increase its predictive capacity and its analytical intelligence. The data used as input for the neural network comes from the agency for the control and supervision of consumption and power generation of photovoltaic solar panel installed in the city of Douala in Cameroon.  Table 1 shows the input dataset for a 5 years period used for forecasting photovoltaic power generation.

The variation of these parameters allows forecasting the photovoltaic generation for a long period. Considering these factors, it is possible to forecast the power generation of solar panel in a city to satisfy the electrical demand.

#### 2.1.2. MATLAB

The software used in this study is MATLAB (Matrix laboratory). It is used to process information automatically through analysis algorithms. In addition, MATLAB also ensures the implementation of the artificial intelligence and deep learning methods in order to more efficiently analyze the data implemented in the system. In this study, the version of MATLAB used is MATLAB R2020b 64 bit. This is one of the most recent versions of this software to optimize calculations. In this study, we implemented our forecasting through neural network adjustment application, support vector machine, and PSO algorithm in MATLAB.

#### 2.1.3. Computer

The simulations of this work were done on a DELL computer with following characteristics: core i5, 3.1 GHz processor, 8 GB RAM, Windows 10/64 bits.

### 2.2. Methods

In this study, an ANN model with SVM and PSO was used as a tool for forecasting solar generation in the city of Douala. Therefore, this model accurately modelled the nonlinear behavior of climate change data and variations of energy demand. Real-time data used include solar irradiance, temperature, humidity, wind speed, and load consumption which are the best input parameters to predict solar generation.

**Figure 1:**
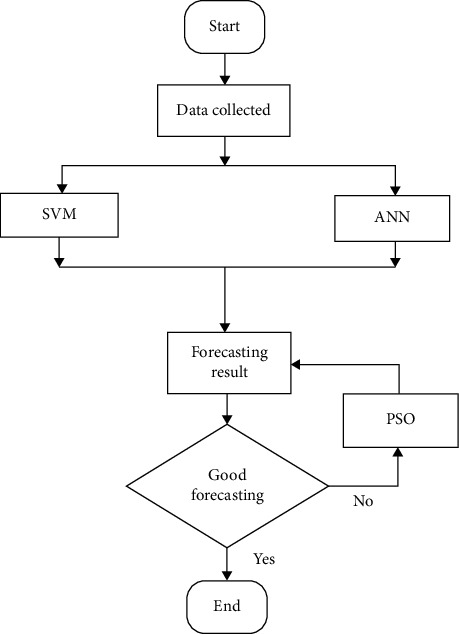
Flowchart of the proposed hybrid intelligent method.

In this model, solar irradiance represents the solar power in an area, and its expression is kw/m^2^. Temperature is the heat degree of the climate, humidity is the cold degree of climate, and wind speed represents the velocity of wind of the locality. Load consumption concerns the consumption of consumers in the city. The data are collected for the 5 years period from January 2016 to December 2020 in the city of Douala in Cameroon. The challenge is to predict the solar power generation to supply consumers with the aim to avoid any outages. This work can forecast the solar generation using a hybrid machine learning method made of the ANN, SVM, and PSO algorithm. The flowchart of the proposed method is described in Figure 1.

In Figure 1, the proposed model consists to initialize the collected data in the machine learning system made of the hybrid model with the neural network and support vector machine. Knowing that the dataset comprises information of climate change and load consumption, the training of the neural network can be done with effectiveness. The continuous step of training, testing, and validation is done in the memory of the neural network for global implementation of the hybrid method. The simultaneous working of these algorithms allows obtaining forecasting results depending from the training data in the dataset. A first forecasting test is done with the aim to evaluate the effectiveness of the computational algorithms. The technique can then calculate the error and evaluate the reliability of the forecasting. When the error is high, a particle swarm optimization algorithm is trained to update the forecasting results. The work is validated when the desired results with high precision is obtained.

#### 2.2.1. Artificial Neural Network

**Figure 2:**
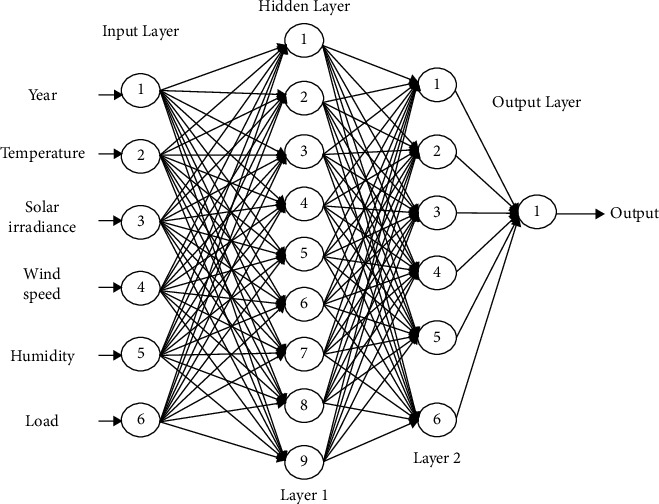
Structure of the multilayer ANN model.

The structure of the multilayer artificial neural network (ANN) model is shown in Figure 2.

Considering the input data, the hidden layers, and the weight, the obtained output data is presented in the following equation:

(1) $$\begin{array}{c} y=f\left(\overset{n}{\underset{i=1}{\sum }}x_{i}w_{i}\right)\text{,} \end{array}$$

where *x*_*i*_ is the input data, *w*_*i*_ is the weight, and *y* is the output data of the artificial neural network.

The following equation gives the sigmoid function as the activation function of the ANN model.

(2) $$\begin{array}{c} f\left(x\right)=\frac{1}{1+e^{-x}}\text{.} \end{array}$$

**Figure 3:**
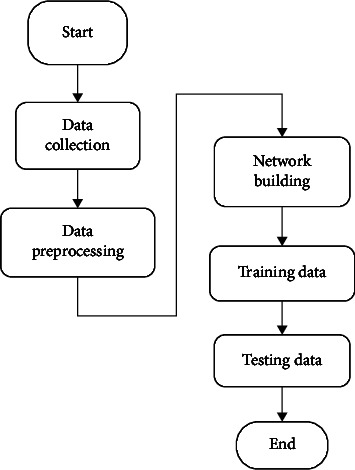
Functional flowchart of the ANN model.

The ANN model implemented in this study is structured through several successive stages ranging from data collection to model validation.  Figure 3 shows the flowchart of the operation of the ANN model proposed in this study.

This model makes it possible to forecast power generation of renewable energy sources through a succession of stages. First, the ANN model needs to collect data. These data come from the supervision and control center of photovoltaic generation in the city of Douala. Second, the model starts data preprocessing to check the input dataset. This step directly follows data collection and allows regularization and standardization of the data collected. The third step is to design and build the neural network through an architecture consisting of neurons, input layers, hidden layers, output layers, activation functions, and learning algorithms. Fourth, the model should start training of the collected data. This step adjusts the input data to converge on the desired target by changing the weights. This step also makes it possible to properly choose the learning algorithms of the ANN model. Finally, the testing data step validates the learning of the neural network. The activation function trains the neural network based on the input data to produce a base associated with the target outputs. Once the neural network has adjusted the data, it forms a generalization through a relationship between the inputs and the outputs and can be used to generate the outputs for which the inputs have not been trained. The data training is done through the training of Levenberg–Marquardt. The number of hidden neurons is 100 neurons which is a significant number of neurons for data accuracy.

#### 2.2.2. Support Vector Machine

**Figure 4:**
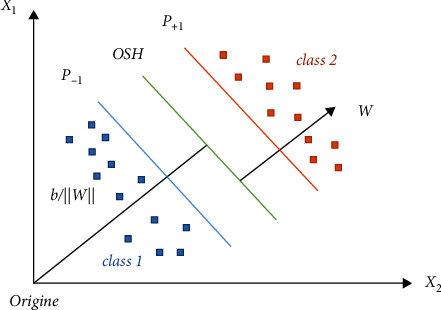
Support vector machine.

The support vector machine (SVM) was developed by Vladimir Vapnik in the years 80 using statistical tools, optimization, and neural network. The SVM is a powerful deep learning method used for supervised classification and linear regression applications. In this work, SVM is used for prediction of solar power generation. Also, the support vector machine can effectively label data using a separating hyper plane as it is described in Figure 4.

The support vector machine is based on hyperplane which can separate the training data between two subgroups (+1 and −1). Among all planes of separation between two classes, there is an optimal space plane and the maximal element training for machine learning generalization.

The negative and positive hyper planes are given in the following equations.

(3) $$\begin{array}{c} w^{T}x_{pos}+w_{0}=+1\text{,} \end{array}$$

(4) $$\begin{array}{c} w^{T}x_{neg}+w_{0}=+1\text{.} \end{array}$$

Then, the subtracting the two previous equations gives the following equation.

(5) $$\begin{array}{c} \frac{w^{T}\left(x_{pos}-x_{neg}\right)}{\left\|w\right\|}=\frac{2}{\left\|w\right\|}\text{.} \end{array}$$

The purpose of the SVM is to optimize the margin between positive and negative hyperplanes. Using the kernel method, it is possible to transform the original features into space with higher dimension. The separation is done by linear hyperplanes as shown in Figure 4.

The SVM performance can be optimized by selecting tuning parameters as the *C* parameter which is the regularization parameter and gamma parameter. We obtain a small margin hyperplane by using a higher value of *C* with the aim to classify correctly the training data. Then, larger margin is obtained with smaller value of *C* parameter, so it will permit misclassification points in training data. In the same time, the gamma parameter is used for the RBF kernel. If the gamma is higher, the hyperplane boundary is tighter. A large gamma parameter leads to higher bias and lower variance.

**Figure 5:**
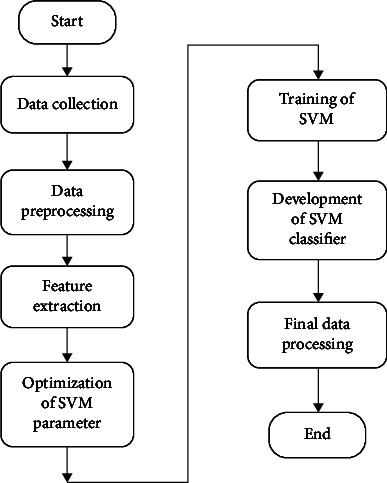
Flowchart of the SVM model.

The flowchart of the SVM model is presented in Figure 5.

First, the data are collected from the electrical agency and climate agency. Then, data preprocessing presents the technique of data mining used for preprocessing of information. Feature extraction consists to extract the essential parameter as temperature and solar irradiance. The optimization of the SVM parameter consists to estimate the accuracy of learning the SVM model by adjusting the error. The training of SVM allows building the SVM model using the optimization of parameters by the number of sample. The training of the model permits to train the inputs data as they can converge to targets. The development of SVM classifier consists to define the training values allowing forecasting the future variables. It can illustrate the learning and training of SVM for optimization of parameter, the development of SVM classifier, the test, and the validation of the SVM model.

The final data processing describes the development of algorithm which can choose and select the suitable forecasting of the SVM classifier.

#### 2.2.3. Particle Swarm Optimization

The particle swarm optimization (PSO) is a computational technique developed by Kennedy and Eberhart in 1995. The PSO is based on social behavior of birds. The concept of swarm used in PSO consists of collective behavior of particles in their environment. A number of particles are used to initialize the PSO algorithm; then, each particle can move to converge toward the best potential region with best solutions. Each particle detains a memory function and adjusts its path using the experiences of other particles.

Each particle can follow the path based on *P*_best_ and *G*_best_ which are, respectively, the best particular position and the best global position of the swarm. They also have two vectors, *X*_*i*_ and *V*_*i*_, respectively, the position vector and the velocity vector of particle.

The following equation gives the velocity vector of the PSO.

(6) $$\begin{array}{c} V_{i}^{t+1}=V_{i}^{t}+c_{1}r_{1}\left(P_{best}-X_{1}^{t}\right)+c_{2}r_{2}\left(G_{best}-X_{i}^{t}\right)\text{.} \end{array}$$

A random solution can initialize the PSO algorithm for optimal solution by updating the particle position as presented in the following equation.

(7) $$\begin{array}{c} X_{i}^{t+1}=X_{i}^{t}+V_{i}^{t+1}\text{,} \end{array}$$

where *c*_1_ and *c*_2_ are the social coefficients, *r*_1_ is the random number generated in a loop from 0 to 1, *X*_*i*_^*t*+1^ is the next position of particle, and *X*_*i*_^*t*^ is the previous position of particle.

**Figure 6:**
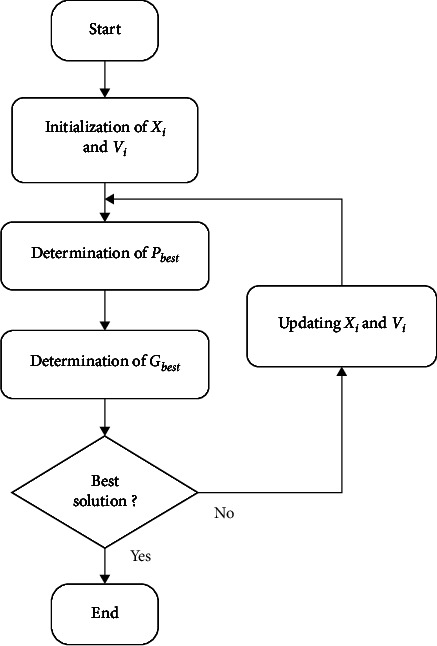
Flowchart of the PSO algorithm.

Figure 6 shows the flowchart of the PSO algorithm.

The algorithm starts with the initialization of position vector *X*_*i*_ and velocity vector *V*_*i*_ of particle. Then, the algorithm determines the best particular position *P*_*best*_ and the best global position *G*_best_ of the swarm. At the end, the algorithm verifies the value of solution; if the value is the best solution, it validates the solution. If it is not the best solution, the algorithm must update *X*_*i*_ and *V*_*i*_ to restart the work.

#### 2.2.4. Evaluation Metrics

The model performance acceptability criteria depend on error factors such as the mean square error (MSE) in equation (8), the root mean square error (RMSE) in equation (9), the mean absolute percentage error (MAPE) in equation (10), and the mean absolute error (MAE) in equation (11). Moreover, in equation (12), the error gives the difference between the forecast and the real data. In addition, the correlation coefficient (*R*) in equation (13) also makes it possible to show the precision of the model during the forecast. Greater is the *R*, better is the forecasting.

(8) $$\begin{array}{c} MSE=\frac{1}{n}\overset{n}{\underset{i=1}{\sum }}{\left({\hat{y}}_{i}-y_{i}\right)}^{2}\text{,} \end{array}$$

(9) $$\begin{array}{c} RMSE=\sqrt{\frac{1}{n}\overset{n}{\underset{i=1}{\sum }}{\left({\hat{y}}_{i}-y_{i}\right)}^{2}}\text{,} \end{array}$$

(10) $$\begin{array}{c} MAPE=\frac{1}{n}\overset{n}{\underset{i=1}{\sum }}\left|\frac{{\hat{y}}_{i}-y_{i}}{y_{i}}\right|*100\%\text{,} \end{array}$$

(11) $$\begin{array}{c} MAE=\frac{1}{n}\overset{n}{\underset{i=1}{\sum }}\left|{\hat{y}}_{i}-y_{i}\right|\text{,} \end{array}$$

(12) $$\begin{array}{c} Error=\overset{n}{\underset{i=1}{\sum }}{\hat{y}}_{i}-y_{i}\text{,} \end{array}$$

(13) $$\begin{array}{c} R=\sqrt{1-\frac{\overset{n}{\underset{i=1}{\sum }}{\left({\hat{y}}_{i}-y_{i}\right)}^{2}}{\overset{n}{\underset{i=1}{\sum }}{\left({\hat{y}}_{i}-y_{i}\right)}^{2}}}\text{,} \end{array}$$

where ${\hat{y}}_{i}$ is the predicted value, *y*_*i*_ is the real value, and *n* is the sample size.

The RMSE defined in equation (9) is a measure of differences between actual values and forecasted values using the model. The RMSE also represents the standard deviation between predicted values and real values. Moreover, the RMSE can be a good measure of precision, but only compare predicting errors of different models for a particular variable and not between all variables. Then, we also used MAPE and MAE defined, respectively, in equations (10) and (11) to evaluate and compare different model performances.

## 3. Results and Discussion

**Figure 7:**
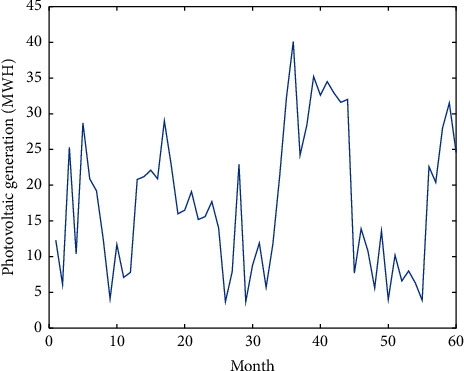
Photovoltaic power generation data.

**Figure 8:**
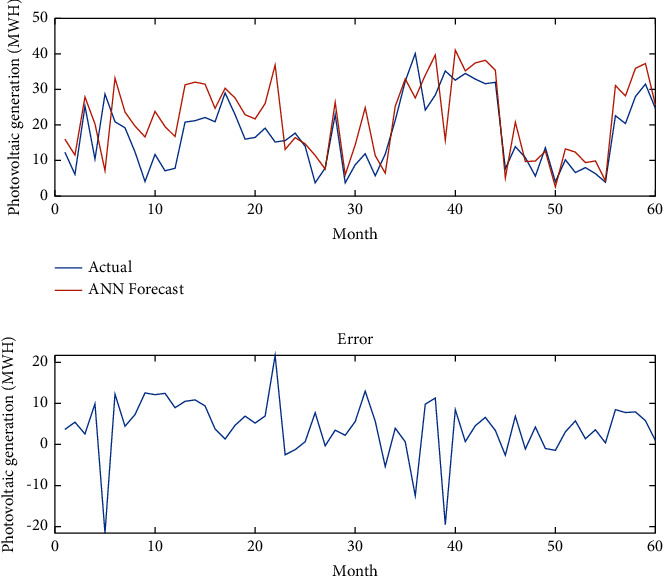
Forecasting using the ANN model.

**Figure 9:**
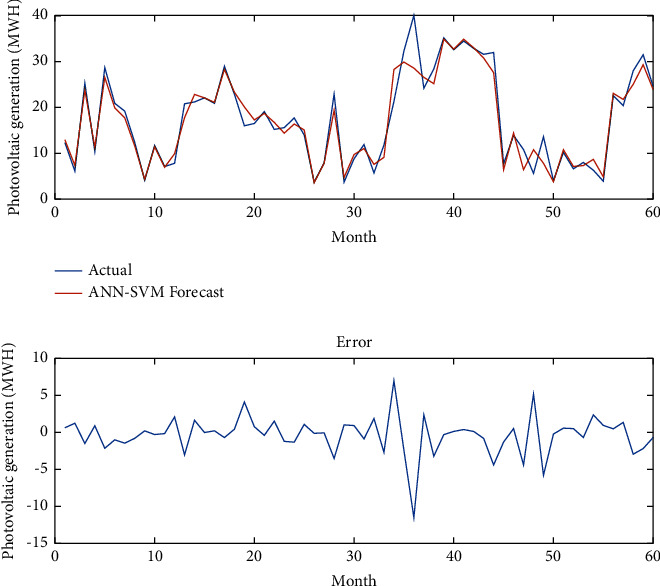
Forecasting using the ANN-SVM model.

**Figure 10:**
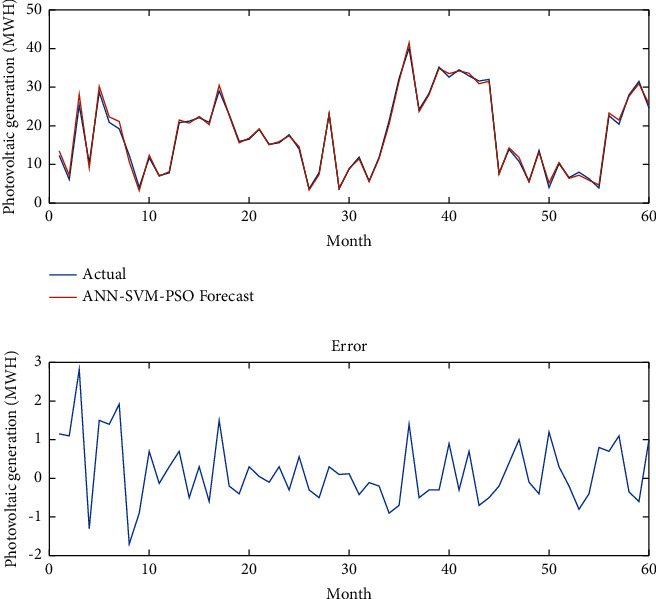
Forecasting using the ANN-SVM-PSO model.

Figure 7 presents photovoltaic power generation data over last 5 years (September 2016–December 2020) in the city of Douala. Figures 8–10, respectively, give the forecasting using the ANN model, the ANN-SVM model, and the ANN-SVM-PSO model.

In Figure 8, the ANN model gives good prediction of photovoltaic generating considering weather conditions and constraints. The prediction almost follows the real data for the prediction period. It can also be seen that the error is low during this period, but it can reach high value for certain month.

In Figure 9, the ANN-SVM model can forecast the power generation using the climatic factors. Moreover, this model gives a better result than the single ANN model during the prediction horizon because it can consider more individual parameters for the prediction process. It can also be observed that the error is to better minimize and that allows to validate this model for the power generation forecasting.

Considering the inputs data such as temperature, humidity, solar irradiance, load, and wind speed, they proposed a novel predictor using a novel hybrid ANN-SVM-PSO model. The implementation of the hybrid ANN-SVM-PSO model using MATLAB helps to obtain the results of the performance of the model based on the input data consisting of irradiance, wind speed, temperature, humidity, and load. Using these input data, the model gives us the forecasting of photovoltaic power generation through neural network adjustment.

In Figure 10, the ANN-SVM-PSO model forecasts the power generation, and this result is better than all previous models implemented in this study. The forecasting perfectly follows the real data which explain the effectiveness of the proposed model. Moreover, the forecasting error is greatly minimizing. The proposed model is effective for this time series solar power prediction considering any factors in the environments.

In Figure 10, the analysis of the behavior between the actual output data and the forecasting output data show that the value of the correlation coefficient (R) is close to 1 which means that the forecast data and the actual data are very close. The real data are obtained from the monitoring center of the city of Douala and the forecasted data are obtained from the simulation algorithm of MATLAB. The actual photovoltaic power generation and the forecast photovoltaic power generation considering all the environmental factors and the model have been implemented over 5 years which means 60 months.

**Figure 11:**
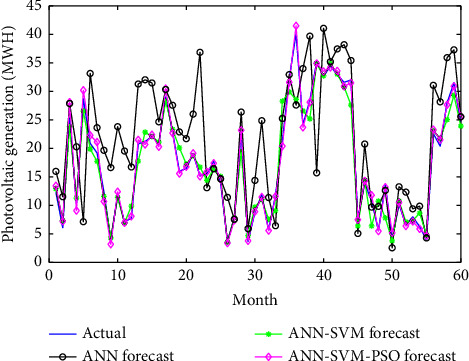
Result of forecasting models.

In Figure 11, we present the comparison of forecasting values for models implemented in this study.

It can be seen that the hybrid intelligent ANN-SVM-PSO model gives better results of forecasting with a low error compared with the previous models. In Figure 11, the forecast power generation using the ANN-SVM-PSO model is closer to actual generation. These results are verified using the correlation coefficient which is close to 1. These forecasting results are satisfactory and verify the accuracy of obtained results.

Depending on climate change, the solar generation will access 35 MWh in the next months. The increase of input values with strong correlation can theoretically give low correlation with the forecasting level, but hybrid model based on historical sequences can improve the prediction using its own sequence of processing.

A summary of the results of forecast models from our study is shown in Table 2.

So, some comparison tests were carried out with the aim to validate the efficiency of our novel hybrid intelligent model. The experimental results using the MSE, RMSE, MAPE, MAE, and *R* allow evaluating the performance of the baseline models and the proposed model. The intelligent model of this study satisfies the requirements and has better results of forecasting considering precision and processing time. First, we have process with the ANN model; second, we used hybrid ANN-SVM model, and finally, we constructed an intelligent hybrid model ANN-SVM-PSO. Our combined method and the previous method have been compared to show the advantages of the proposed model. It can be observed that the proposed method gives better results with low errors compared with other models. In that case, performance of the ANN combined with the SVM and PSO algorithm is better than single ANN model and the hybrid ANN-SVM model.

Moreover, a comparative study with the literature is shown in Table 3.

The comparison of the results shows that this model is better than the models that exist in the literature. With respect to the results, most of the evaluation metric results of our model are higher than those of the baseline methods of the literature. Furthermore, clustering on datasets of the proposed method can significantly improve performance compared with traditional machine learning methods. Using the proposed model, the regression coefficient is better because the forecast generation is close to the actual generation.

## 4. Conclusion

The management of photovoltaic power generation is important because it is impacted by several parameters that can influence the power supply to consumers. In addition, forecasting is a vital operation for the management of energy generation systems, in particular photovoltaic generation. Many research studies have been done to increase the accuracy of renewable resources forecasting with multiple methods and solutions. This study presents a novel hybrid intelligent method for forecasting solar photovoltaic generation in the city of Douala which is affected by environmental influences such as solar irradiance, temperature, wind speed, and load consumption. The implemented model is based on the artificial neural network (ANN), the support vector machine (SVM), and the particle swarm optimization (PSO) algorithms. To verify the efficiency of this model, we used the MATLAB software for the forecast over a period of 5 years of generation of solar photovoltaic plants. Using a simple ANN model, the RMSE is 6.7240 and *R* is 0.9554. The combination of the ANN and SVM presents the RMSE and *R* as 6.3449 and 0.9714, respectively. In order to improve these results, we implement a novel hybrid intelligent ANN-SVM-PSO model. The new values of RMSE and *R* are 3.8693 and 0.9984, respectively. The results obtained in this study show the efficiency of this novel intelligent model compared to those in the literature because the forecast error factors are very low and the correlation coefficient is higher. At our knowledge, it is the first paper which can predict photovoltaic generation using a hybrid intelligent machine learning model.

## Figures and Tables

**Table 1:** Dataset.

Month	Temperature (Celsius degree)	Solar irradiance (kWh/m^2^)	Wind speed (m/s)	Humidity (%)	Load (MWh)
01	23	154.25	67.75	36.2	93.1
02	22	173.25	72.25	38.5	93.6
03	22	154	66.25	34	95.8
04	26	184.75	72.25	37.4	101.8
05	24	184.25	71.25	34.4	97.3
06	24	210.25	74.75	39	104.5
07	26	181	69.75	36.4	105.1
08	25	176	72.5	37.8	99.6
09	25	191	74	38.1	100.9
10	23	198.25	73.5	42.1	99.6
11	26	186.25	74.5	38.5	101.5
12	27	216	76	39.4	103.6
13	32	180.5	69.5	38.4	102
14	30	205.25	71.25	39.4	104.1
15	35	187.75	69.5	40.5	101.3
16	35	162.75	66	36.4	99.1
17	34	195.75	71	38.9	101.9
18	32	209.25	71	42.1	107.6
19	28	183.75	67.75	38	106.8
20	33	211.75	73.5	40	106.2
21	28	179	68	39.1	103.3
22	28	200.5	69.75	41.3	111.4
23	31	140.25	68.25	33.9	86
24	32	148.75	70	35.5	86.7
25	28	151.25	67.75	34.5	90.2
26	27	159.25	71.5	35.7	89.6
27	34	131.5	67.5	36.2	88.6
28	31	148	67.5	38.8	97.4
29	27	133.25	64.75	36.4	93.5
30	29	160.75	69	36.7	97.4
31	32	182	73.75	38.7	100.5
32	29	160.25	71.25	37.3	93.5
33	27	168	71.25	38.1	93
34	31	218.5	71	39.8	111.7
35	31	247.25	73.5	42.1	117
36	32	191.75	65	38.4	118.5
37	35	202.25	70	38.5	106.5
38	30	196.75	68.25	42.1	105.6
39	36	363.15	72.25	51.2	136.2
40	30	203	67	40.2	114.8
41	35	262.75	68.75	43.2	128.3
42	34	205	29.5	36.6	106
43	37	217	70	37.3	113.3
44	39	212	71.5	41.5	106.6
45	39	125.25	68	31.5	85.1
46	33	164.25	73.25	35.7	96.6
47	40	133.5	67.5	33.6	88.2
48	39	148.5	71.25	34.6	89.8
49	35	135.75	68.5	32.8	92.3
50	37	127.5	66.75	34	83.4
51	37	158.25	72.25	34.9	90.2
52	30	139.25	69	34.3	89.2
53	31	137.25	67.75	36.5	89.7
54	39	152.75	73.5	35.1	93.3
55	32	136.25	67.5	37.8	87.6
56	34	198	72	39.9	107.6
57	38	181.5	68	39.1	100
58	32	201.25	69.5	40.5	111.5
59	34	202.5	70.75	40.5	115.4
60	40	179.75	65.75	38.4	104.8

**Table 2:** Forecast errors of forecasting models.

Model	MSE	RMSE	MAPE (%)	MAE	R
ANN	45.2134	6.7240	7.695	2.665	0.9554
ANN-SVM	40.2587	6.3449	4.524	1.244	0.9714
ANN-SVM-PSO	14.9721	3.8693	3.32	0.867	0.9984

**Table 3:** Comparative study with literature.

Model	MSE	RMSE	MAPE (%)	MAE	R	Authors
ANN model	90.8078	9.5293	95.65	3.254	0.8511	[28]
Artificial neural networks and an analog ensemble	85.236	9.2323	92.32	3.122	0.8724	[31]
Wavelet transform model	65.2585	8.0782	75.44	2.855	0.9133	[32]
Genetic algorithm optimized hidden Markov model	52.6982	7.6358	65.47	2.544	0.9375	[42]
Multidirectional search optimization algorithm	47.5442	6.8952	61.02	2.142	0.9524	[43]
Multiheaded convolutional neural networks	39.2515	6.2651	54.25	1.857	0.9755	[44]
ANFIS model	34.6934	5.8901	44.23	1.566	0.9814	[45]
ELM model	30.4836	5.5212	31.05	—	0.9824	[47]
ANFIS-PSO model	17.2225	4.1525	26.17	1.356	0.9857	[48]
Nonlinear autoregressive neural network	—	—	—	0.94	—	[49]
The hybrid ACO and PSO model	—	—	3.513	—	—	[50]
ANN-SVM-PSO model	14.9721	3.8693	3.32	0.867	0.9984	Writers

## Data Availability

The data used to support the findings of this study are included within the article.
